# Ethnic Stereotype Formation and Its Impact on Sojourner Adaptation: A Case of “Belt and Road” Chinese Migrant Workers in Montenegro

**DOI:** 10.3390/ijerph18189540

**Published:** 2021-09-10

**Authors:** Alexander S. English, Xinyi Zhang, Adrian Stanciu, Steve J. Kulich, Fuxia Zhao, Milica Bojovic

**Affiliations:** 1 Department of Psychology and Behavioral Science, Zhejiang University, Hangzhou 310027, China; 2 SISU Intercultural Institute, Shanghai International Studies University, Shanghai 200083, China; xinyizhang513@gmail.com (X.Z.); blueye77@163.com (F.Z.); milica.bojovic.93@outlook.com (M.B.); 3 GESIS—Leibniz Institute for the Social Sciences, 200083 Mannheim, Germany; Adrian.Stanciu@gesis.org

**Keywords:** cultural learning, Chinese migrant worker, cultural distance, psychological, adaptation, sociocultural adaptation, stereotype formation

## Abstract

Ethnic stereotypes are cognitive markers that are formed and modified because of intercultural contact with a new cultural group. There is now much empirical evidence that explicates how stereotypes of ethnic groups can impact individuals’ acculturation experiences. However, what is unknown is how previously nonexistent ethnic beliefs are formed as a result of contact with the local culture. One hundred and seventy-four (*N* = 174) overseas Chinese construction workers were contacted through the Chinese Consulate in Montenegro and agreed to participate in the present study. The online questionnaire asked workers to describe Montenegrin majority members in terms of five characteristics. These traits formed the stereotype markers that were classified as positive, neutral, or negative. Sojourners also answered questions that measured perceived cultural distance, social exclusion, knowledge of Montenegrin culture, feelings of social exclusion, and their sociocultural adaptation. Results show that both positive and negative stereotypes are correlated with less social exclusion and better socio-cultural adaptation. Perceived cultural distance, knowledge of host culture and length of stay was mediated by stereotypes on adaptation outcomes. Interestingly longer sojourn did not lessen the type of stereotype, nor did it reduce cultural distance. Contribution to the stereotype literature and practical understanding of how Chinese sojourners see majority members will be discussed.

## 1. Introduction

In the uncertain global economic climates, many would welcome the opportunity of a new job offer. However, the offer at hand comes with the challenging condition of relocating for a few years to a country that you have limited or no prior knowledge of. You do not know what the local culture entails, nor do you have any preexistent information about the local population. As a short-stay immigrant in the country (sojourner) you will nonetheless have to find ways of coping with challenges such as experiencing periods of homesickness or communicating effectively with locals; the job entails that you will have to make choices on how to acculturate and adapt in some ways to local realities [1]. This is not an uncommon proposition in our age of global mobility, but some cognitive and affective dynamics, especially aspects related to prior knowledge or pre-departure perceptions, are understudied [2]. Intercultural sojourns originating from or between non-western countries are also understudied. This paper offers some key answers as to how sojourners come to “know” their local communities and how that knowledge might impact their adaptation process.

The literature extensively documents extensively affective and behavioral changes that sojourners must undergo when crossing cultures [3,4]. In contrast, the cognitive domain of cultural adaptation has been insufficiently investigated and research needs to clarify such processes as cognitive heuristics (stereotypes) when acculturating to new places [5]. For instance, what is cultural adaptation like for vastly different ethnic groups with no prior knowledge of the country where they will be working in? We are interested in exploring the idea that sojourners who have little or no previous cultural knowledge might accurately form ethnic stereotypes which could lead to better psychological and sociocultural adaptation. In this present study, we focus on the case of Chinese migrant workers in Montenegro and their reported stereotypes of the local majority members.

**Figure 1:**
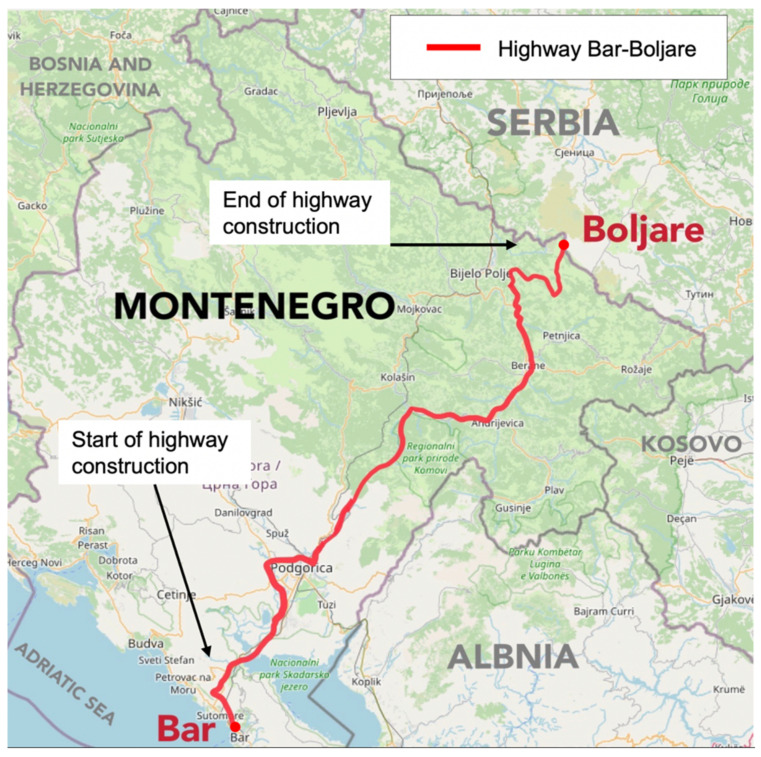
Map depicting the CBI project in Montenegro. Note. Red colored line show the Montenegrin highway construction project conducted by Chinese sojourners. As the project developed, Chinese workers were relocated farther away from the local population.

China launched the Belt and Road Initiative (BRI) as a way to enhance regional connectivity and collaboration with neighboring and strategic partner nations and increase involvement in international investment and infrastructure development in more than 60 other nations. One important BRI-related pact is with the Central and Eastern European countries (CEEC) and Montenegro was one of the first countries of those 16 to become an active partner. Agreeing on strategic development needs, approximately 3000 Chinese workers were relocated in Montenegro for approximately 36 months to build 165 km of roads, 48 tunnels, and 107 bridges and viaducts with the intention of connecting Montenegro with bordering Serbia (see Figure 1). The intended construction connects the two nations, and as the project progressed the workers had to move farther away from major cities in self-sustaining worker encampments. The project created a unique context of intercultural relations wherein a group of sojourners live, work, and speak their native language while being de facto in a foreign country of which they have little to no previous knowledge. This paper investigates the cognitive heuristics-adaptation of Chinese sojourners in Montenegro and whether this mediates their cultural adaptation.

### 1.1. Cultural Adaptation among Sojourners

Cultural adaptation is an outcome in the acculturation process—the process by which immigrating individuals change or adapt aspects of the self-due to experiencing contact with the host culture [4]. Traditionally in the literature, the migrant is seen as an agentic actor who faces the choice between how much of the origin culture to maintain and how much of the host culture to adopt [1]. Depending on individual (or group) choice and unconscious actions, an acculturation orientation follows. For example, the migrating individuals tend to choose separation by maintaining their origin culture and discarding elements of the host culture, integration (keep origin and adopt host), assimilation (drop origin and adopt host), and marginalization (drop both origin and host).

Adaptation means that migrating individuals have identified effective ways to cope with the initial distress and novelties related to the new living conditions, norms, or environment of the new country [3,6,7]. Cultural learning is the motor that triggers these processes leading to more adaptation; it is oftentimes referred to as “getting to know” a culture [3,8]. Migrating individuals are exposed to novel cultural information and therefore learn new ways of being, thinking, or affectively relating to the reality that prevails in their host country [9,10].

Sojourners are voluntary migrants who relocate to other countries for a predetermined period [4,11]. They can be tourists, exchange students, or skilled workers (expats). Sojourners experience the process of learning a new culture similarly to other migrant typologies: they seek out new culturally relevant information, engage in intercultural learning opportunities, and observe and imitate the majority members [8,12].

The literature has concerned itself almost exclusively with sojourners (or migrants in general) who possess prior knowledge about their host countries and the local majority group. Tourists choose a destination because they know what the country offers regarding landscape or “tourist attractions”. International students decide on a country because they are aware of its education system, culture, or traditions. Expats are also offered choices regarding relocation and must similarly seek to gain basic knowledge on how to function in the new business (and if they have families, also the social and educational) culture and pick up some survival local language.

Another category of sojourners is workers (“blue-collar”, laborers), who due to the nature of their contracts, generally must live, work, and interact amongst themselves (in company provided camps/enclaves); for example, the Chinese construction workers of the BRI employed in Montenegro. They may not have completely “chosen” to make this move but rather were assigned to do so by their companies. Having limited opportunities to interact with the local culture and population and usually possessing no prior knowledge of the culture nor of the population, in what ways does their cultural adaptation unfold? It is worth noting that this condition is not what is normally theorized in adaptation models, whether individuals to choose to adopt a separation strategy or another orientation, most acculturation frameworks assume or imply individuals or groups have agency.

### 1.2. Ethnic Stereotype Formation Is the Missing Link in Cultural Adaptation

Ethnicity is generally regarded to be at the forefront of individuals’ identity during intercultural contact since it represents an extension of the culture that people belong to (i.e., Chinese, Montenegrins) [3,4,13]. Understanding one’s or others’ ethnicity is central for a sojourner’s cultural adaptation. Not only does ethnicity assist a person in dealing with the challenge of living in two cultures where two ethnic groups are the majority [1], but it also determines how the majority ethnic group in the host culture treats the person [14]. Under the increasingly prominent condition of bi-ethnic individuals (people belonging to two cultures), those whose identity draws a greater sense of fulfillment from the host culture than the origin culture report better cultural adaptation in terms of wellbeing or feeling of belonging [15]. One explanation is that the beliefs people associate with their own and others’ ethnicity lead into appropriate behavior and feelings towards self and other or the ethnic group that the other represent [16] (also see Social Identity Theory). Having no prior knowledge of the local ethnic group is an obvious gap in the sojourner’s ability to navigate the new-to-them reality. In this case, we argue, ethnic stereotype formation is a precondition for cultural adaptation [5].

Ethnic stereotype formation is a socio-cognitive event that occurs due to a process of culture learning. Ethnicity is a learned ascription—through socio-cultural interactions individuals become aware of both their ethnic belonging and of what other ethnic groups exist in society as well as what each stands for in aspects such as status and agency [17]. The formation of ethnic stereotypes can be traced back to early socialization [18] and to first encounters with members of a novel ethnic group [5].

Stanciu and Vauclair [5] proposed that through cultural learning, migrating individuals incorporate the stereotype-relevant information they learn in host cultures into preexistent stereotypical beliefs—the so-called stereotype accommodation hypothesis [19,20]. Migrants can adjust, for example, the stereotype they previously held about the majority ethnic group towards an understanding of the culture of the local population in their host country. Crisp and Turner [21] described the ability of people to form accurate understandings of the ethnicity of others despite limited or inaccurate preexistent knowledge of what the ethnicity stands for. Cultural contact can associate with ethnic stereotype formation and reconciliation of any inaccurate preexistent stereotype content (stereotype formation and stereotype accommodation are used interchangeably in this paper).

Sojourners with prior knowledge of the majority ethnic group in their host countries “only” need to learn what the local beliefs about the group are [22,23]. We would thus expect sojourners with previous cultural knowledge to adapt more effectively, yet this relationship has not yet been examined in the literature [24]. For instance, the stereotype accommodation hypothesis does not explicitly state that previous cultural knowledge implies more effective adaptation, but it nonetheless indicates that possessing knowledge of the ethnic category would lead to less information to process and expedite the socio-cognitive adaptation [20]. Sojourners with no previous knowledge of the majority ethnic group are likely to seek out relevant cultural knowledge and try to understand what the group stands for and what values and beliefs are important to them. We would expect sojourners in these situations to experience a greater cognitive burden in the acculturation process resulting in greater acculturative difficulties or stress.

Further factors which influence stereotype formation among sojourners include cultural distance and duration of stay in the host country [5]. Cultural distance is the difference between two cultures in terms of overt aspects such as language, traditions, or food as well as covert aspects such as beliefs, norms, or values [25,26]. Two major conceptualizations of cultural distance exist: objective cultural distance refers to national indices covering domains such economics and cultural values [27] while perceived cultural distance (PCD) refers to the relationship between subjective indicators that individual sojourners encounter [28,29]. Generally, the greater cultural distance one feels, the more acculturative challenges one experiences in his or her cultural adaptation [3,30]. Nonetheless, some studies have not found such effects [31] including one within the Chinese context [32]. The stereotype accommodation hypothesis states that greater cultural distance implies a large pool of inconsistent/novel stereotype-relevant information that immigrants must navigate and therefore decide on whether to integrate these into preexistent beliefs.

The duration of stay in the host country also affects stereotype formation by facilitating opportunities to learn about novel cultural information. A longer stay in the host country is typically an indicator of a better sociocultural adaptation [4]. With a longer stay, sojourners have opportunities to gain more information inconsistent with any of their preexisting stereotypes, resulting in a modification of the initial stereotype [5,20]. Similarly, with a longer stay, sojourners become aware of social categories that are entirely new to them and some of the stereotypical beliefs associated with these categories (e.g., understanding the concept of Manelisti in Romania [33]).

### 1.3. The Present Research

The present research examines whether ethnic stereotype formation mediates the relationship between adaptation of sojourner Chinese skilled workers in Montenegro and their psychological and sociocultural adaptation outcomes. No previous research has addressed these themes or considered these intergroup relations. A reason for doing this study is the typical assumption in the research that migrants possess at least some prior knowledge of the host culture and local population. The case of Chinese skilled workers in Montenegro is unique in that they have likely neither migrated by choice, nor have had any cultural preparation or pre-knowledge of the context they are sent to, and are also bound by the nature of their contract to work and live almost exclusively amongst themselves.

In the case of large-scale Chinese construction projects in BRI countries, workers are mostly confined to their work location (even unique from “typical expat” compounds where “white-collar” workers can choose what levels of society of the local culture to mix with). Focusing on the construction site and project progress, a physical relocation away from the local culture and population is often necessitated, so that these Chinese sojourners experience few opportunities for intercultural contact with locals. To check this assumption, data for the present research was collected during the third year of the construction project, so the Chinese workers in Montenegro had at least in theory some opportunities for intercultural contact from their prolonged stay [5].

Ethnic stereotype formation is ideally a topic for longitudinal research endeavors. However, research adopting a cross-sectional approach is likewise acceptable and sometimes even desired [34]. Here we operationalize ethnic stereotype formation in ways similar to classical research on stereotype content [35], as we seek to meet the criteria for emic cultural research [36]. We suggest that collecting free descriptions is one way to approximate the formation of stereotype content toward a previously unavailable ethnic category. It is important to note that stereotype valence (positive attitude or negative attitude) is outside of our research focus or design. Though that would be pertinent for research following the contact hypothesis [37], in this context, extensive contact is limited. Therefore, we consider the collection of ethnic stereotypes (evidence that they have been formed and can be articulated) as expressing the density/richness of stereotypical traits associated with encountering a novel ethnic category [38].

The first set of hypotheses concern the formative effects of the acculturation experience on ethnic stereotypes held by Chinese sojourners in Montenegro. Under the term acculturation experience, we here understand processes that involve (a) perceiving the cultural distance between the origin and host culture, (b) knowing the local culture, and (c) the duration of stay in the host country. Given the two vastly different ethnic groups, we expect that:

**Hypothesis 1a** **(H1a).** *Greater perceived cultural distance will be associated with both more positive and more negative-connoted ethnic stereotypical traits.*

**Hypothesis 1b** **(H1b).** *Having more knowledge of the local culture will be associated with more positive and more negative-connoted ethnic stereotypical traits.*

**Hypothesis 1c** **(H1c).** *A longer stay in the host culture will be associated with more positive and more negative-connoted ethnic stereotypical traits.*

Next, we hypothesize that the formed ethnic stereotype will be a predictor of Chinese sojourners’ adaptation in terms of psychological and socio-cultural aspects. Psychological adaptation indicates the absence or resolution of stress-related symptoms caused by the acculturation process, such as having good mental health and feelings of being in the right place [3]. Here, we operationalize psychological adaptation as the absence of perceived social exclusion [6]. Sociocultural adaptation is the ability of migrating individuals to operate in the new country in terms of dealing with novelty across domains of life, which include weather, food, and societal norms [6]. We predict:

**Hypothesis 2a** **(H2a).** *More ethnic stereotypical traits (a better formed ethnic stereotype) will be associated with a greater absence of perceived social exclusion.*

**Hypothesis 2b** **(H2b).** *More ethnic stereotypical traits (a better formed ethnic stereotype) will be associated with better sociocultural adaptation.*

Further hypotheses for this study concern the indirect effects that the acculturative experience can have via the formed ethnic stereotype on the cultural adaptation of Chinese sojourners. If knowing who the local population is and what they allegedly stand for is indeed pivotal in the cultural adaptation process, then we expect a replication of the existent literature on the effects of the acculturative experience on sojourner adaptation [3,4]. That is, we predict that:

**Hypothesis 3a** **(H3a).** *A greater perceived cultural distance will be indirectly associated with greater feelings of social exclusion and worse sociocultural adaptation.*

**Hypothesis 3b** **(H3b).** *More knowledge of the host culture will be indirectly associated with a greater absence of social exclusion feelings and with better sociocultural adaptation.*

**Hypothesis 3c** **(H3c).** *A longer duration of stay in the host culture will be indirectly associated with a greater absence of social exclusion feelings and with better sociocultural adaptation.*

## 2. Methods

### 2.1. Participants and Procedures

Data was collected as part of a larger study on mutual cultural adaptation and perceptions on Chinese in Montenegro and Montenegrin sojourners in China. Questionnaires were distributed in the participants’ native language and data was collected through an online survey. Given the difficulty of recruiting this sample, workers were contacted through the Chinese Embassy in Montenegro in December 2017. An embassy official directed the fifth author to the one of the head engineers at the Head Camp of the China Road and Bridge Corporation (Montenegro Branch in Podgorica, Montenegro). After several step-by-step confirmational emails to ensure our university project proposal and aims (IRB board approval and to confirm the project did what it aimed to do), the head of the China Road and Bridge Corporation (Montenegro Branch) granted us access via social media contact distribution to Chinese workers who were in Montenegro currently working on the highway project. Head engineers recruited their team members from their various sites to complete the survey online. No personal information was collected about team members or the head engineer. The purpose of this project was to understand perceptions of Montenegrin life and cultural adaptation.

Before participants started the questionnaire, researchers provided a digital informed consent form to ensure they were aware that their answers were fully protected. In total, there were 174 Chinese (average age was 29.08, *SD* = 6.19, 90.2% male, average stay in host culture was 18.9 months *SD* = 12.6). Of the entire sample, 91.4% of them reported working on the construction site, while the rest reported “other”.

### 2.2. Analytical Procedure and Power Analysis

**Figure 2:**
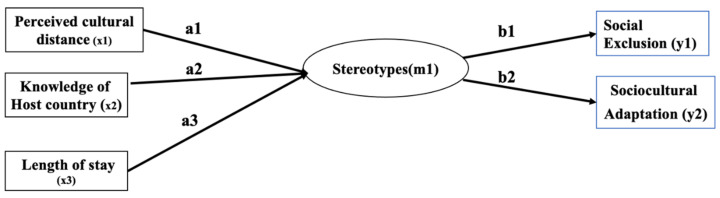
Theorized mediation model, where Xs are the predictors, M is the mediator and Ys are the outcome variables.

Unless otherwise specified, we used a significance level of α = 0.95 and all tests were performed double-sided. All analyses were done using SPSS and AMOS v.20. We had a three-stage analytical procedure that was applied to the latent stereotype model, which includes both negative and positive stereotypes. First, Hypotheses 1a–c were examined through calculating simple Pearson correlation coefficients. Second, Hypotheses 2a,b tested the direct effect of PCD, knowledge of host and length of stay on stereotypes. Third, for Hypotheses 3a,b a mediational model tested how the latent stereotype variable mediated the relationship between our antecedent variables (PCD, knowledge of host, length of stay) and dependent variables (see Figure 2). To identify evidence for model fit, we used the following thresholds: α with an associated *p* > 0.05, Comparative Fit Index (CFI) value is greater than 0.90 and the Root-mean-square Error of Approximation (RMSEA) is between 0.08 and not less than 0.06 [39]. Direct and indirect effects (*a*_s_ and *b*_s_) are estimated with bootstrapping (5000 iterations) with 95% bias-corrected confidence intervals.

In Structural Equation Modeling (SEM), power analysis has generally focused on RMSEA [40] and involves the evaluation of the probability that one can detect effects that are viable in the population [41]. This measure of model of fit (or misfit) is observed per degree of freedom based on the fit function [42]. For the convention of power gradients above 0.80, the model fit should have a RMSEA of 0.80 or below. Given this basic threshold, our model and sample were adequate to find small and medium effect sizes.

### 2.3. Measurement

Demographics: Participants first completed basic demographic information (age, gender) and background information (length of stay and purpose of sojourning to Montenegro).

Ethnic stereotypes: Participants freely wrote down five open-end associations related to Montenegrins in Mandarin Chinese [35,43]. Three Chinese-English bilinguals independently coded these open-ended answers as negative (−1), neutral (0), or positive (1) stereotypical traits. For example, “polite” and “smiling” were rated 1, “quiet” was rated 0, and “lazy” and “stubborn” were rated −1. The results presented here are based on a consensual coding by three independent researchers with *k*s > 0.80. Finally, two stereotype indices were created by summing each participant’s positively- and negatively connoted traits. Higher scores on the stereotype measurement implies more diverse content of stereotypes along the positive or negative dimensions.

Perceived cultural distance, sociocultural adaptation, and knowledge of host country: Perceived cultural distance and sociocultural adaptation were measured using the Perceived Cultural Distance (PCD) Scale and the Sociocultural Adaptation (SCA) Scale [6], both including 12 items concerning questions on, for example, climate and food and drinks. Participants responded based on a 7-point Likert scale (from 1—very similar, to 7—very different) (PCD; α = 0.88). The SCA items asked how difficult it was for them to adjust in terms of the 12 aspects, also based on a 7-point Likert scale (from 1—very difficult, to 7—very easy) (SCA; α = 0.91). Participants were also asked about the level of their knowledge of host country in terms of five characteristics of Montenegro including: geography, history, politics, economy, and culture [24]. Their answers were recorded on a Likert scale (1—nothing, 7—a lot). Item examples are: “What do you know about the country’s geography and “What do you know about the country’s politics?” A compiled score of all the items was created (α = 0.88).

Perceived social exclusion: Participants indicated how excluded they felt in general by the local population in their host country [24]. Five items were provided, and the responses were recorded on a Likert scale (1—strongly disagree, 7—strongly agree). Item examples are: “I feel as if most of the people in my host country do not want to mix with me” and “I have the impression that most of the people in my host country prefer to avoid me”. A composite score was created over the answers on the five items (Cronbach *α* = 0.81).

## 3. Results

As it can be seen in Table 1, the positively connoted traits of “friendly” (47.1%) and “warm-hearted” (36.2%) as well as “polite” (20.7%) were the most endorsed positive traits and surpassed negatively reported traits. Chinese sojourners described only one negative trait, which is that Montenegrins are allegedly “lazy” (21.3%). All other negative traits can be considered as exceptions to the rule as their frequency is much lower. Also, noteworthy is that the tenth most frequent positive trait, “optimistic,” was shared by a greater number of Chinese sojourners (8.0%) than the second most frequent negative trait, “stubborn,” which was shared by 7.5% of the sample. Overall, the Chinese seem to perceive their host country nationals mostly in a positive manner with one striking exception—lazy.

### 3.1. Test of Hypotheses 1—Correlations

Table 2 provides an overview of descriptive analysis and correlation between the variables in this study. Both positive stereotypes and negative stereotypes were negatively related to perceived cultural distance (*r* = −0.23, *p* < 0.01; *r* = −0.32, *p* < 0.001) and to social exclusion (*r* = −0.29, *p* < 0.001, *r* = −0.26, *p* < 0.001). Meanwhile, they were positively related to SCA (*r* = 0.22, *p* < 0.01, *r* = 0.23, *p* < 0.01). Only the positive stereotype factor was positively related to knowledge of the host country (*r* = 0.20, *p* < 0.01).

### 3.2. Test of Hypotheses 2—Direct Effects

**Figure 3:**
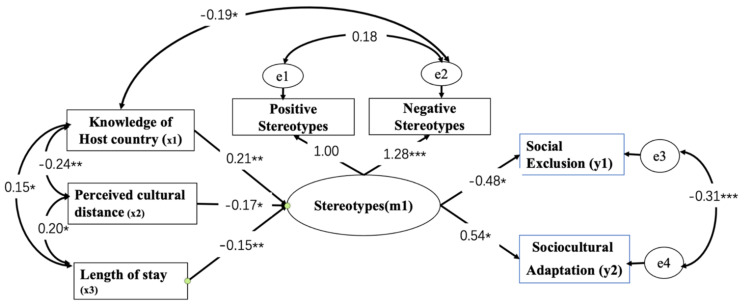
Latent stereotype structural model (χ^2^(8) = 13.092, *p* = 0.109, CFI = 0.976; RMSEA = 0.061). Note: All reported estimates are standardized. * *p* < 0.05, ** *p* < 0.01, *** *p* < 0.001. Using model modification indices, knowledge of host country and negative stereotype were a significant path that was included in the final model (discussed more below).

Our results (Figure 3) reveal that knowledge of the host country positively predicted stereotypes (*B* = 0.206, SE = 0.072; *p* = 0.010). Perceived cultural distance negatively predicted stereotypes (*B* = −0.171; SE = 0.066; *p* = 0.013). Length of stay negatively predicted stereotypes (*B* = −0.151, SE = 0.065; *p* = 0.005). This finding suggests that workers who lived in the host country longer and had less PCD also reported more stereotypes towards majority members. Those with more knowledge of Montenegro had better articulated stereotypes towards locals.

Meanwhile, stereotypes predicted social exclusion (*B* = −0.478; SE = 0.191; *p* = 0.030), and sociocultural adaptation (*B* = 0.536; SE = 0.327; *p* = 0.020). Workers with a more nuanced stereotype felt less excluded and more adapted in Montenegro.

### 3.3. Test of Hypotheses 3—Indirect Effects

For the following factors, all indirect effects are reported in Table 3.

Knowledge of Host Country to Adaptation Outcomes

Knowledge of host country was indirectly and negatively associated with social exclusion (*B* = −0.098, *p* =0.035, CI 95% [−0.261, −0.006]). It was indirectly and positively associated with sociocultural adaptation (*B* = 0.110, *p* = 0.049, CI 95% [000, 0.302]).

Perceived Cultural Distance to Adaptation Outcomes

Perceived cultural distance was indirectly and positively linked with social exclusion (*B* = 0.082, *p* = 0.025, CI 95% [0.010, 0.227]). It was indirectly and negatively associated with sociocultural adaptation (*B* = −0.092, *p* = 0.043, CI 95% [−0.272, −0.001]).

Length of Stay to Adaptation Outcomes

Length of stay was indirectly and positively linked with social exclusion (B = 0.072, *p* = 0.007, CI 95% [.022, 0.192]). It was indirectly and negatively linked with sociocultural adaptation (*B* = −0.081, *p* = 0.022, CI 95% [−0.191, −0.005]).

## 4. Discussion

The present research investigation addressed how stereotypes can mediate the acculturation relationship between antecedent variables and adaptation outcomes for temporary work migrants. Analyses confirmed Hypotheses 1–3. Stereotypes were associated with sociocultural adaptation and social exclusion. The mediation model also confirmed that stereotypes mediated the relationship between knowledge of host culture, perceived cultural distance, length of stay, and adaptation outcomes.

In sum, this study used data from a group of Chinese workers who had little prior knowledge of Montenegro prior to sojourning, yet during their stay they were asked to characterize the local people in numerous ways. Regardless of the type of stereotype (positive or negative), possessing some stereotypes impacted the relationship between three antecedent variables (cultural distance, cultural knowledge, and length of stay) and adaptation outcomes (social exclusion and sociocultural adaptation). Novel in and of itself, this study also attempted to identify how a minority group formed stereotypes toward a majority host group, which has not been addressed in the acculturation literature [44]. We discuss the contributions, limitations, and practicalities in further detail below.

### 4.1. Prior Knowledge Is Needed in Establishing Ethnic Stereotypes

The results of the mediation analysis corroborate past evidence on migrants’ cultural adaptation while it adds extra layers of complexity. Indirect effects of perceived cultural distance, for instance, perfectly reproduced findings that a greater perceived cultural distance was associated with a greater feeling of social exclusion as well as a lesser sociocultural adaptation [45]. In the literature up to this point, feelings of exclusion or a lack of sociocultural adaptation have been attributed to more negative images of the majority population [46]. However, the novelty of our study is the finding that this can happen irrespective of the ethnic stereotype associated with the local host population. Chinese sojourners felt socially excluded and reported less sociocultural adaptation in Montenegro regardless of if they had a more nuanced stereotype, either positive or negative, toward Montenegrins. Therefore, it seems probable that a greater cultural distance is not automatically associated with lesser adaptation via more negative opinions of the local host population. It is more likely that a greater cultural distance means lesser knowledge about who and what the local host population stand for [5], and as a result, the migrating individual gains a sense of misplacement and exclusion.

Another important finding was the association between knowledge of the host country and negative stereotypes. In our model fit testing, modification indices suggested including this pathway. The greater the knowledge of the host country, the fewer negative stereotypical attributes. This seems to go slightly in the direction of the contact hypothesis which states that maintained contact with a group will result in decreasing prejudice against that group [47]. While this finding is very preliminary, exploratory analyses could consider the role of the contact hypothesis further.

We were unable to find direct associations between duration of stay in the host country and indicators of cultural adjustment (social exclusion and sociocultural adaptation) despite existing literature predicting such associations [48]. This is an immediate consequence of the type of sojourners we examined—Chinese workers in Montenegro who, due to the nature of their contracts, had limited opportunities for contact with the local host culture and population.

As we stated in the Introduction, the minimal assumption in the current literature has been that migrants (sojourners, long-term migrants, etc.) have at least some opportunities to interact and connect with members of the local population and therefore learn about their culture. In contrast, by being enclosed with their ethnic group, working and going about social activities amongst each-other, Chinese sojourners in Montenegro had restricted access to otherwise normally-existing cultural learning channels. As highlighted in Table 1, we found that generally Chinese sojourners generally reported favorable, positive stereotypes about Montenegrins and little uniformity in regards to negative stereotypes. This itself is remarkable given their relocation assignment. This result also supports a recent qualitative study on Chinese working abroad in Indonesia and Sudan [49]. Researchers found the working environment to be highly similar to that in China and that their living environment was a shared dormitory with other Chinese and a full-time chef recruited from China. This Chinese enclave also represented a simulated Chinese home where workers never lose touch with their homeland but might feel excluded from host culture communities. In contrast, Western expats also share a similar “bubble type” experiences; however; we might expect cross-cultural adjustment experiences to be more diverse and less constrained to one’s home-country community.

Nonetheless, the mediation analyses revealed one striking finding, namely, the duration of stay in the host country was indirectly associated with indicators of cultural adjustment when we considered Chinese workers’ stereotypical beliefs about their local host population. This finding is remarkable in the sense that, whether positive or negative, a stereotype about a Montenegrin explained that the effects of social exclusion and sociocultural adaptation were due to a longer stay as opposed to more perceived cultural distance.

Interestingly, ethnic stereotypes did not become more positive or negative as a result of inter-ethnic culture learning. This might be due to evidence provided in the sample that the same stereotype is shared to a greater extent by many sojourners even with longer stays in Montenegro. Perhaps the lesser heterogeneity and cohort effects influence their perceptions of the local population. Past research may have focused more on individual-level determinants, but it is possible that this robust set of shared stereotypes could be a characteristic of collectivist groups [50]. This contributes to the stereotype accommodation theory, as it seems to be a degree of sharing of stereotypical traits—a result of learning about the local cultural group.

Considering the effects of possessing knowledge of the host country, results revealed a direct association with sociocultural adaptation but no association with feelings of social exclusion. Admittedly, in retrospect, one might have argued that knowledge of a country’s climate or politics cannot impact one’s feelings of being excluded or not. This is because the elements of the association (predictor and outcome) correspond in fact to two distinct theoretical frameworks [3]. Knowledge of the host country should “in theory”, and here “in practice”, be associated with a greater “doing well” (sociocultural adaptation)—the more information migrants possess about the reality in the host country, the better their adaptation will be in terms of being able to navigate that reality (speaking the language, adapting to the local climate, etc.). Feelings of exclusion, on the other hand, refer to skills in dealing psychologically (feeling well) with stress due to living abroad [1], which should again “in theory”, and “in practice” here, be predicted by perceived cultural distance, among other factors. We did find, however, indirect and positive associations with both indicators of cultural adjustment when we considered the ethnic stereotypes held by our participants toward the local Montenegrins.

The indirect effects of knowledge of the host country on feelings of being socially excluded and sociocultural adaptation were exclusively via positive stereotypes and in both cases their ultimate impact was positive. This is a counter-intuitive finding—why should knowing about a country’ politics, for instance, matter in a process of forming stereotypical knowledge about an ethnic group? We suggest that knowing about the host country is to also know about who its members are and what they stand for. In the context of migration, forming stereotypical content about a novel, or previously unknown, social category resembles in many ways the process of forming stereotypical content in early socialization [51]. People rely on external kinds of information from others in developing schemata or patterns that can be subsequently used in categorizing and implicitly stereotyping others [52].

Although Chinese sojourners have developed both positive and negative stereotypical beliefs about the host Montenegrin population, possessing knowledge about the host country was only associated with positive beliefs. It is still unclear as to why this was the case; there are three possible explanations. The first explanation implicates the origin culture of the present study’s mostly-homogenous participants—the Chinese culture, which is known to be conflict aversive, to deal with problems indirectly [53,54] and to pursue politeness and harmony [55,56]. As a consequence, the study’s finding might be applicable only to the present sample (or similar Chinese cultural background groups). This study showed that the more knowledge of Montenegro Chinese sojourners possessed, the greater was their willingness to express only positive beliefs associated with the local population. In prejudice research, this would be interpreted as participants tending to suppress any genuine negative associations with a target group, despite negative associations being present [57].

The second explanation is that only positively connoted stereotypes are active in psychological mechanisms that transfer existent knowledge of the host country into cultural adaptation and feelings of inclusion into that country. This explanation stems from research on the contact hypothesis which holds that, for example, increased contact with members of other ethnic groups can lead to more positive beliefs associated with them and less discrimination toward them [58]. Contact need not be direct to impact beliefs and behavior toward an interacting individual or group but can also be by extension (knowing someone who directly knows a member of the other group) or imagined [59]. It is probable that, in the case of Chinese sojourners who work and live together, members of the group contributed to stereotypes of the local host population. For instance, young new arrivals could be acquiring trait information from other colleagues and not from the host culture. Research on ‘cultural brokers’ or liaisons suggest that new arriving expats often get host-culture information from other expat colleagues, social contacts at bars or social events, and social media sources [60].

The third, and currently the more intuitive explanation, is that the result is due to chance, a social desirability bias, or face-giving given that the data was collected via links to Chinese embassy officials. It is possible that construction workers responded in a way that alluded to positive experiences and that only after observational or longitudinal evidence could demonstrate a more conclusive set of results. Though it raises interesting questions that further research should explore, we advise prudence in reading too much into the finding.

### 4.2. Contributions and Implications

This study contributes to a large gap in the stereotype literature by examining how the concept of stereotype accommodation can manifest and influence short-term migrants’ (sojourners’) acculturation. We argue that migrants integrate the stereotype-relevant information that they learn in their host culture into preexisting stereotypes and that these perspectives on inter-ethnic stereotypes then deepen their knowledge of host culture society. Literature has noted that stereotypes can lead to detrimental effects for migrants, yet in our study, we find that not only do both positive and negative stereotypes lead to more sociocultural adaptation, but also to fewer feelings of being excluded.

What may not have been adequately differentiated in past literature are the goals or purposes of specific migrant groups: the “immigrant” disposition has generally been assumed to be the preferred condition of expats, and typically, acculturation is seen as an ongoing cultural learning process by adding new culturally relevant knowledge to existing knowledge [8]. Yet certain types of cultural exchanges in globalization and new international strategies such as the BRI may be drawing our attention to specific or new types of sojourners, the study of which might advance theories on antecedents and outcomes of cultural adaptation or sojourner adjustment profiles.

In practical terms, this research investigation provides understandings of migrant workers’ lives in a unique cultural context. Chinese BRI has been seen with mixed reactions on the international stage and in varied local contexts. On one hand, Chinese projects provide much-needed technical expertise and support for poor or developing countries, but this development can come at a cost to some of these nations if this incurs debts or substantial loan payments back to China in the future. In addition, some countries’ media have reported that the BRI has not brought employment to local workers because most Chinese projects self-fund, self-hire, and bring their equipment from China.

There is clearly a broad range of responses to the BRI. Though these issues are noted, press reports from Montenegro seem to have been more positive than negative—BRI projects like this one in Montenegro do bring huge economic advantages and conveniences to the country (such as the first cross-nation highway). Based on these contributions, trading, and manufacturing between other CEEC countries are expanding and improving relations with China—this BRI project has certainly helped expand bilateral relations and also bring more Montenegrins to China (whose adjustments are also being studied).

## 5. Limitations and Conclusions

One important limitation in this study is that it is a cross-sectional study, which limits the testing of some potentially interesting causal predictions (e.g., concerning the relationship between acculturation variables, stereotype formation and adaptation outcomes). The nature of this post-hoc, single-set of self-reported data also does not allow us to test causal relationships proposed in the Stanciu and Vauclair stereotype accommodation model, nor does it yet allow us to reach robust conclusions on stereotype formation, both of which are interesting lines of inquiry for future research.

Second, the data is self-reported only; and might reflect how respondents say they are adapting, not how they might actually be adapting in practice. In other words, there may be a cognitive bias at play (e.g., “If I say everything’s fine, then everything really will be fine”). One obvious answer is follow-up qualitative, preferably ethnographic, research within the same cohort to juxtapose “adaptation as imagined” with “adaptation as reality” which might include watching the day-to-day interactions of the construction workers with Montengrins.

Third, since the only way to collect this data was through social media links provided through official channels at the Chinese Embassy in Montenegro, it is possible that there is a response bias or social desirability effects. While data from a rare temporary migrant population was collected, this sample is also limited in its representativeness for other sojourning groups (e.g., international students and expats).

A factor that was not investigated or considered in this study is the role of education and training in stereotype formation. It is common practice in international expatraite assignments for companies to offer predeparture training or post-arrival programs for employees to learn about local cultures, we have no knowledge if some or all the sojourners in this study already had some or any cultural training. Previous studies have highlighted the importance of this factor [61,62], so future designs could consider how to include this.

Finally, another consideration is the possibility that, assuming the workers are not too familiar with the Montenegrin culture, as their migration was rather unplanned, their stereotype characteristics might be a measure capturing the initial stages of stereotype formation, given the lack of previous knowledge. A longitudinal study to measure trajectories across individuals would provide evidence to support this claim. In sum, we hope this exploratory study can be viewed with caution but also open some important lines of research to explore the development of stereotypes among varied types of sojourners in diverse conditions to capture perception processes toward host country populations.

This present study sought to examine the experiences of Chinese construction workers building a major highway that is already connecting Montenegro with Serbia/Europe and reflecting generally positive bilateral Sino-Montenegrin relations. Our study showed that regardless of positive or negative stereotypes, the possession of some salient stereotype will lead to better adaptation and less social exclusion. Cultural distance, knowledge of host culture and time in Montenegro were all shown to be mediated by stereotypes and had a direct effect on their overall experiences. In conclusion, this study should be seen as a building block in stereotype formation literature that complements the growing number of studies in non-western cultural settings.

## Figures and Tables

**Table 1:** Stereotype content of Montenegrins mentioned by the Chinese sojourners.

	Stereotype Content	Frequency	%
	Positive traits		
1	友好/友善 (Friendly)	82	47.1
2	热心肠/热情 (Warm-hearted)	63	36.2
3	有礼貌 (Polite)	36	20.7
4	漂亮 (Beautiful)	26	14.9
5	生活悠闲/惬意/休闲 (Relaxed)	21	12.1
6	好客 (Hospitable)	20	11.5
7	微笑 (Smiling)	16	9.2
8	注重生活/懂生活/享受生活 (Enjoying life)	16	9.2
9	善良/和蔼/亲切 (Kind)	15	8.6
10	乐观 (Optimistic)	14	8.0
	Negative traits		
1	懒/懒散/懒惰 (Lazy)	37	21.3
2	固执/倔强 (Stubborn)	13	7.5
3	穷 (Poor)	11	6.3
4	迟到/不守时 (Unpunctual)	7	4.0
5	不守信用 (Dishonest)	6	3.4
6	不灵活/一根筋/轴 (Inflexible)	5	2.9
7	吹牛 (Boastful)	5	2.9
8	自大 (Arrogant)	4	2.3
9	不友好 (Unfriendly)	3	1.7
10	自我 (Egoistic)	3	1.7

This table reports the 10 most positive and negative traits after 3 independent coders confirmed positive and negative traits. “Friendly”, “warm-hearted” and “polite” accounted for the majority of positive traits, while negatively, only “lazy” was reported by 20% of the sojourners.

**Table 2:** Descriptive statistics and correlations.

	Variable	α	M	SD	1	2	3	4	5	6	7	8
1	Positive stereotype	~	3.12	1.46	-	0.70 ***	−0.23 **	0.20 **	−0.15 *	−0.29 ***	0.22 **	−0.11
2	Negative stereotype	~	−0.90	1.21		-	−0.32 ***	0.08	−0.22 **	−0.26 ***	0.23 **	−0.11
3	PCD	0.88	4.88	0.91			-	−0.24 **	0.20 **	0.03	−0.20 **	0.03
4	Knowledge of host country	0.88	3.08	0.86				-	0.17 *	−0.08	0.24 **	0.02
5	Length of stay	~	18.94	0.66					-	−0.02	−0.01	0.01
6	Social Exclusion	0.81	2.28	0.98						-	−0.42 ***	0.03
7	SCA	0.91	4.70	0.92							-	−0.07
8	Age	~	29.07	6.19								-

Note. PCD = Perceived cultural distance. SCA = Sociocultural adaptation. Length of stay is measured in months. * *p* < 0.05, ** *p* < 0.01, *** *p* < 0.001.

**Table 3:** Indirect effects of study predictors on social exclusion and sociocultural adaptation in a sample of Chinese sojourner workers in Montenegro.

	Social Exclusion Y_1_	Sociocultural Adaptation Y_2_
Latent Stereotype Model	Indirect effect	Indirect effect
Knowledge of host country (×1)	−0.098 * [−0.261, −0.006]	0.110 * [0.000, 0.302]
Perceived cultural distance (×2)	0.082 * [0.010, 0.247]	−0.092 * [−0.272, −0.001]
Length of stay in host country (×3)	0.072 ** [0.022, 0.192]	−0.081 * [−0.191, −0.005]

Note. Paths are depicted in Figure 2. These results are based on cross-sectional data and thus should be interpreted as correlational rather than causal evidence. Brackets represent values of 95% confidence intervals from a bootstrap test. * *p* < 0.05, ** *p* < 0.01

## Data Availability

Data and material associated with the manuscript are made available on the https://osf.io/quhzp/?view_only=5727e58945bf454ea371f04534e4ac90 (accessed on 20 July 2021).
